# Insiders/outsiders of Canadian disability arts

**DOI:** 10.1017/S2045796023000598

**Published:** 2023-07-19

**Authors:** Eliza Chandler, Sean Lee, Lisa East, Megan Johnson

**Affiliations:** 1 School of Disability Studies, Toronto Metropolitan University, Toronto, ON, Canada; 2 Revision Centre for Art and Social Justice, Tangled Art + Disability, Toronto, ON, Canada; 3 University of Guelph, Guelph, ON, Canada

Disability art is a burgeoning arts sector in Canada that takes the experience of disability as a creative entry point (Chandler, 2019; Chandler *et al.*, 2021). Through creative practice, disabled artists often challenge normative ways of understanding disability by representing their embodiment in agentive, intersectional and nuanced ways that are driven by and authentic to their lived experiences (Abbas *et al.*, 2004, p. 1). As Kelly and Orsini (2016) write, ‘disability cultures and aesthetics can and do transform situated and lived experiences of difference’ (p. 4). Contributing to disability rights and justice, disability arts can confront ableism by asserting the agency, political will, creativity and collectivity of disabled people as culturally vital (Campbell, 2009; Ignagni *et al.*, 2021; Ignagni and Schormans, 2016). Such a cultural redress is significant given that hundreds of thousands of disabled people were institutionalized throughout Canada starting in the mid-1800s; in Ontario, for instance, the first institution was opened in 1876, and the last closed in 2009 (Brown and Radford, 2015; Reaume, 2012). Considered ‘mentally defective’ and ‘incurable’, developmentally disabled people were especially likely to be institutionalized (Brown, et al., p. 26). Institutionalization was partially motivated by the eugenic practice of custodialism that removed disabled people from public spaces, including spaces of arts and culture, because of the belief that their presence was detrimental to the growth of nation building (Malacrida, 2018; Voronka, 2008). There is a well-documented practice of curators coming into these institutions to select artwork created by institutionalized people to exhibit it as ‘Outsider Art’ or Art Brut (Prinz, 2017). Within (and outside of) disability arts cultures, Outsider Art is typically categorized as innocent, non-conscious art most often created by institutionalized artists who were self-taught, situated outside the mainstream art world and whose work was exhibited by non-disabled curators without crediting the artist (Chandler as cited in Chandler, 2017; Nelson, 2016; Prinz, 2017; Sandals, 2016; Swain, 2019). The practice of showing art created by disabled people as Outsider Art mirrored the ableist treatment that disabled people experienced inside of institutions based on the idea that they did not have the capacity to take care of themselves, express themselves, or assert agency in decision-making. As a result of the ways that ableism manifested in circles of Outsider Art, these artists were excluded from standard practices, such as attending exhibition openings or being asked to deliver artist talks (Gorman, 2007; Nelson, 2016). Further, if their artwork was purchased, they did not receive a percentage of the profits, and they were not supported in their professional development (Gorman, 2007; Nelson, 2016).

Legacies of institutionalization persist, impacting disability activism, culture, and politics as well as our resistance. Sean Lee, Director of Programming at Tangled Art + Disability, Canada’s leading disability arts organization, has observed that the mainstream art world continues to position artwork produced by disabled people as a therapeutic tool for healing non-normative bodies and minds rather than as artistic expression and as a vehicle for social change. Such assumptions have ongoing material effects on the lives of disabled people and contribute to the de-professionalization and depoliticization of disability arts. For example, as disability artist Aislinn Thomas (2021) reports, many artists working within day program structures (After deinstitutionalization, social service agencies in Canada started day programs for people with developmental disabilities to gather, learn life skills, and engage in recreational activities such as arts and crafts) still do not have ownership or control over their artwork, choice in subject matter or receive earnings from the sale of their work (para. 4). The continuation of these ableist practices, particularly for artists working within institutions or organizations which mobilize the charity model of disability, has led many disability artists to distance themselves from Outsider Art and its paternalistic, exploitative past. It is often understood that this distancing is necessary for disabled artists to be recognized as professional artists and to gain access to training, funding and exhibition opportunities, which they have been historically denied. As disability studies scholar and artist Rachel Gorman wrote in 2007, ‘…as professional artists, we do need access to continuing professional development. Specifically, we need more workshops organized by artist/activists, rather than by non-disabled-identified artists who want to do away with technique or who push alternative techniques as a substitute for doing the work of translating and adapting technique for the participants’ (p. 50).

Many of the cultural norms within disability culture were established as a way to interrupt legacies of institutionalization and their impact on how disabled people are perceived and subsequently treated. However, in this rush to ‘leave a difficult past behind’ (Hansen, 2018), some of these disability cultural practices – such as the common refrain that disability art is not Outsider Art and the choice to work with arts organizations that are led by disabled people and through a disability politic – have inadvertently discounted experiences of artists who work within trans-institutional spaces, such as day programs (Abbas, 2004; Gold, 2021; Nelson, 2016). In working with artists at Tangled Arts, Lee has noticed that the distinction between art and therapy is less rigid than the disability arts movement would suggest. For example, many artists Lee works with describe first encountering the opportunity to create art and identify as an artist through the very institutional spaces from which mainstream disability arts is seeking distance. For other artists, claiming art as therapy may actually be a political move given a lack of adequate mental health services. For example, mad Black artist Gloria Swain (2019) writes, ‘My depression is political; it is the direct result of anti-Blackness and all the cruelty that has been shown to Black people. My art practice, which animate[s] the connection between madness and anti-Black racism is political and also therapeutic; the two do not cancel each other out’ (para. 2).

The word ‘outsider’ is also a term that is being reclaimed with pride by many artists who are choosing to proclaim their practices as ‘outsider’ not necessarily to align themselves with historic conceptions and practices of Outsider Art but as a way of identifying that they have experienced exclusion and barriers upon trying to enter the mainstream art world. When used in this context, the reclaimed label ‘outsider’ allows artists to break from traditionally held expectations of art and express themselves wholly. Similarly, Kristin Nelson (2016), a professional artist with a disability who does not identify as a disability artist, points out that a focus on identification and intentionality in disability arts definitions may also exclude some artists. Speaking to normative standards within disability arts, Nelson (2016) writes, ‘to be considered a disability artist, one must create work that *represents* one or more disability cultures or one must self-identify as an artist whose *intent* is to advance the professional status of disability artists. The problem with representation is that it limits the kind of art that can be made, dissuading Deaf and disability artists who want to explore art in more abstract ways without jeopardizing their inclusion within the Deaf and disability arts community’ (p. 105). Continuing, Nelson argues that, ‘the problem with requiring artists’ intent for inclusion in the Deaf and disability arts movement is that it inherently excludes artists who, either purposefully or by default of their disability, do not explicitly state their intent’ (p. 105).

Similarly, Lee observes that some disabled artists still experience barriers despite promising systemic changes within Canada’s arts funding agencies. For instance, many project-based grants require a specific way of justifying and articulating the value of a project, which can require language that is inaccessible to artists labelled with or who identify as developmentally disabled or neurodivergent as well as and including those who have not had access to arts education. Disability arts funding tends to favour applications that show promise of making an artistic impact in ways that are recognizable within normative culture. Funding bodies, such as the Canada Council for the Arts, have recognized that some disabled artists are disadvantaged by the application process – sometimes even by the application form itself – and offer application development, but many artists do not have the necessary connections to make use of this assistance.

**Figure 1.**
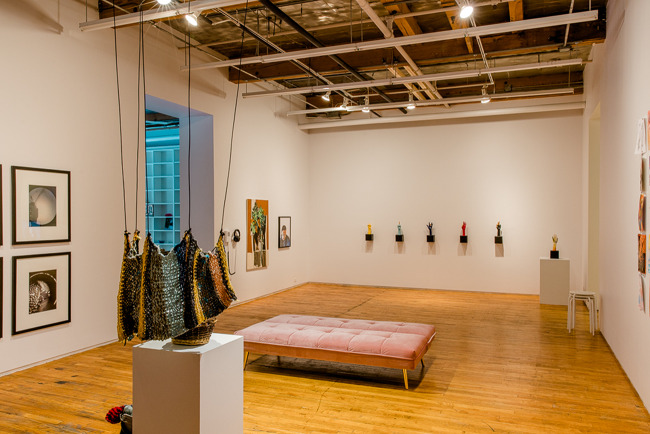
Crip Rituals at Tangled Art Gallery, Installation Shot featuring.

We are attentive to how dominant discourses and practices within disability arts and mainstream arts assume a normative artistic experience, which exclude some disabled artists from accessing material supports, exhibition and other professional opportunities, and even from participation and recognition within disability arts communities. Moreover, we are interested in how some artists with disabilities have started to return to the language of Outsider Arts as a way to push back against these exclusions. We also recognize that meanings and practices of Outsider Arts have shifted and evolved with the contemporary landscape. Clearly, the relationships between Outsider Art, mainstream art and disability art are complex, tenuous and warrant concerted attention. Given this, we call for disability arts to turn towards artists who might be excluded from and by normative cultural practices within our sector while also holding onto the main tenets of disability arts. To explore a way through these tensions, we conclude with a story from Lee’s experience co-curating a recent exhibition at Tangled Arts (Figure 1).

Left to right, Malcolm Corley and Maria Corley’s *Untitled*, Malcolm Corley’s *Untitled #1* and Malcolm Corley’s *Hoodie Self-Portrait*. Photograph by Michelle Peek courtesy of Bodies in Translation: Activist Art, Technology, and Access to Life.

**Figure 2.**
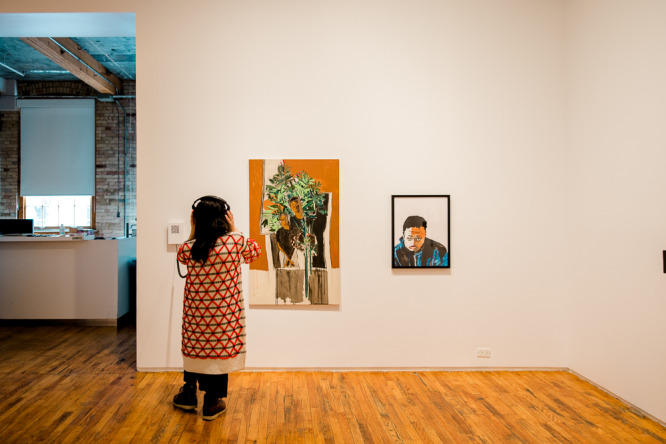
Crip Rituals at Tangled Art Gallery, Installation Shot.

**Figure 3.**
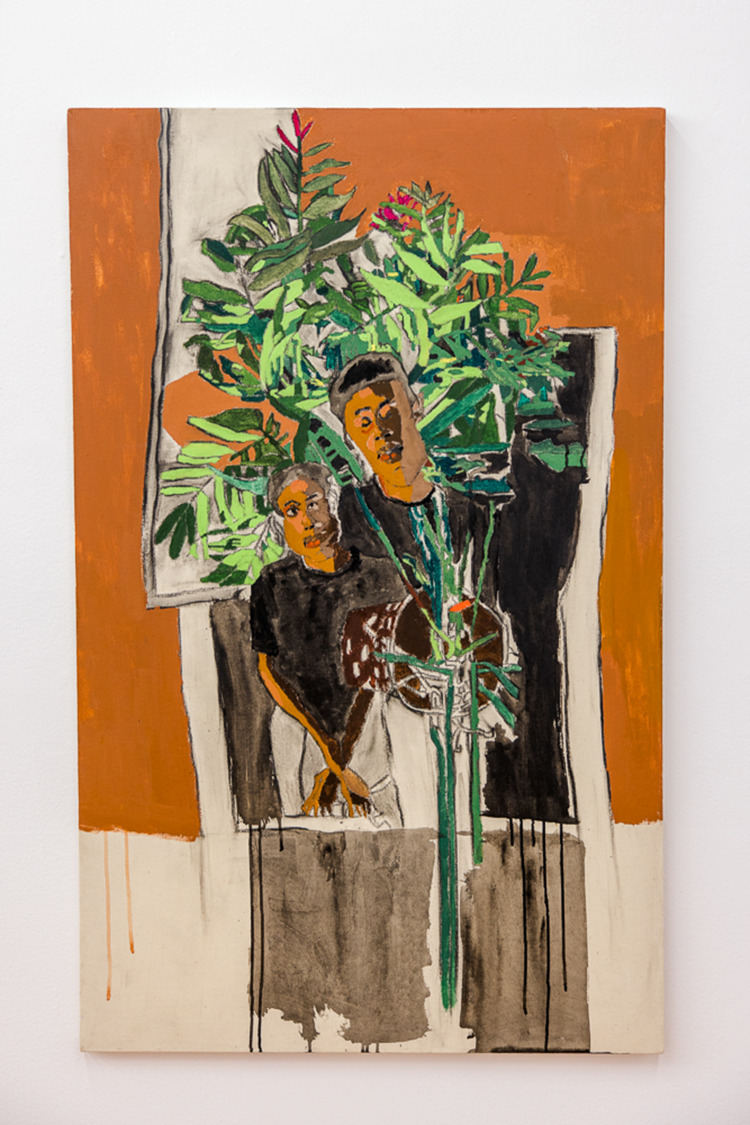
Malcolm Corley, *Untitled #1* 2019.

Tangled Arts has always been a site of political engagement with disability arts and a site for advancing our collective understanding of what a disability justice-oriented approach to art can be. Developing this understanding must include challenging but not abandoning our previous understandings of autonomy. In his role at Tangled, Lee had the opportunity to engage this tension as he worked with the Critical Design Lab to co-curate #CripRitual, an exhibition that featured several disability artists whose practices were grounded in interdependent relationships. Malcolm Corley, a non-verbal autistic artist, was one of the artists featured in this exhibition. The co-curators communicated with Malcolm through his mother and artistic collaborator, Maria Corley, who is skilled at communicating *with* Malcolm not *for* him. When meeting with Malcolm and Maria, curators discussed creative ways for disabled audience members to access his work, ideally through a sensorial modality other than vision. Tangled has these conversations with every artist they work with as part of their commitment to disrupt the ocularcentrism within most disability art galleries – a commitment firmly articulated by disability artist (who also identifies as a non-visual learner) Carmen Papalia (2013). During this conversation, the co-curators discovered that Malcolm often creates his self-portraits while his mother plays piano, another iteration of their collaboration. Building on this collaborative practice, Tangled commissioned Malcolm and Maria to co-create a musical composition that harmonized the sounds Malcolm produces while he creates art together with Maria’s piano playing. Malcolm and Maria’s composition was offered alongside Malcolm’s portrait in #CripRituals as a way for Blind and low vision artists and attendees to access this work. Not only did this composition offer access, but it also unsettled traditional notions of autonomy and independence in art creation (Figures 2 and 3).

Photograph by Michelle Peek courtesy of Bodies in Translation: Activist Art, Technology, and Access to Life.

Gouache on paper, 48 × 30.5 × 2 inches. Photograph by Michelle Peek courtesy of Bodies in Translation: Activist Art, Technology, and Access to Life

Thomas (2021) writes that, ‘interdependence and autonomy are both core values of Disability Arts. Despite this, there is a discomfort with the kinds of interdependency and care relationships that some people labelled with intellectual disabilities need in their lives and artistic practice’ (para. 20). If Tangled were steadfast in their commitment to artistic autonomy as it is conventionally understood within disability arts, they might not have recognized Malcolm and Maria’s collaboration as a practice that belongs in a disability art gallery. However, they recognized that, ‘collaborative practice is real practice. Interdependent practice is real practice’ (Sweeney, as cited in Thomas, 2021, para. 20). The installation of Malcolm and Maria’s work was profoundly moving, and the power dynamics throughout the process felt equitable and egalitarian. As with so many disability artworks, this work foregrounded access in a way that brought together the artistic and the political.

**Figure 4.**
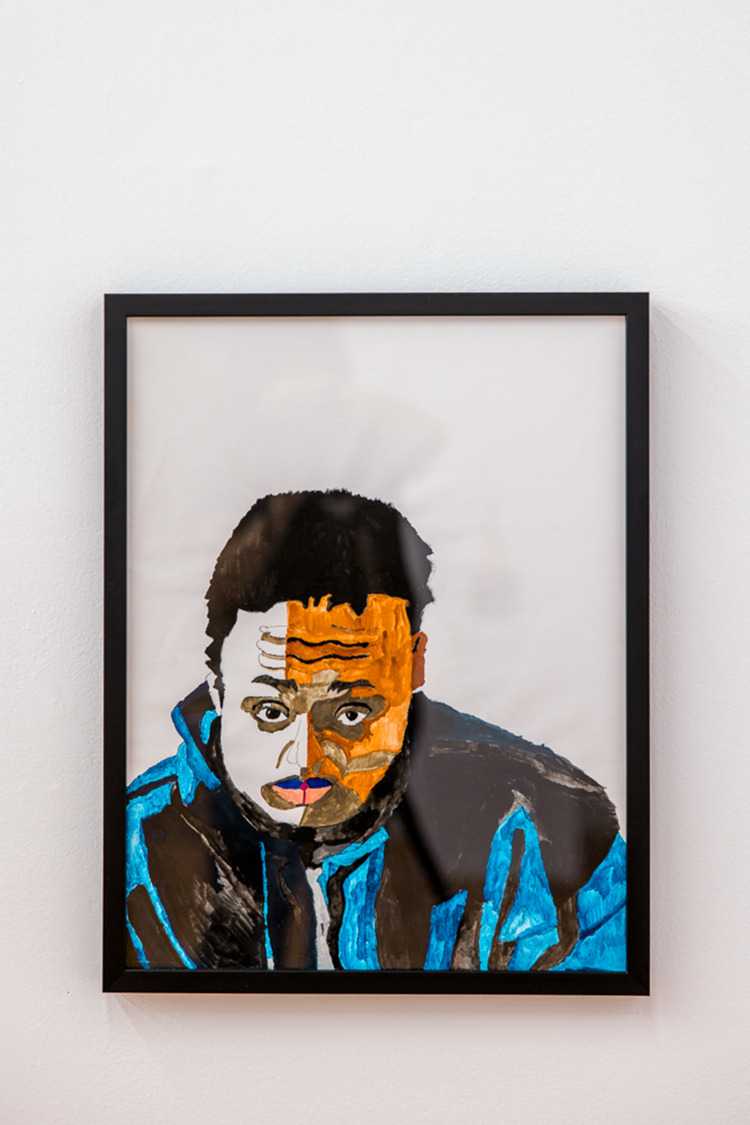
Malcolm Corley, *Hoodie Self-Portrait*, 2018.

As we continue to fight for more just and equitable participation for all artists with disabilities in the arts sector, we are reminded of the ever-moving battle we engage to fight for access and inclusion. As Joseph Grigely writes, ‘this is what makes the gallery a courtroom and a courtroom a gallery: in both, there is a desire for justice alongside a desire for art that does more than just hang on the wall’ (as cited in Cachia, 2023, 5) (Figure 4).

Watercolour on paper, 17.5 × 24 inches. Photograph by Michelle Peek courtesy of Bodies in Translation: Activist Art, Technology, and Access to Life.
